# Comparative Structural and Biophysical Investigation of *Lycosa erythrognatha* Toxin I (LyeTx I) and Its Analog LyeTx I-b

**DOI:** 10.3390/antibiotics14010066

**Published:** 2025-01-10

**Authors:** Amanda Neves de Souza, Gabriele de Azevedo Cardoso, Lúcio Otávio Nunes, Christopher Aisenbrey, Evgeniy Salnikov, Kelton Rodrigues de Souza, Ahmad Saad, Maria Elena de Lima, Jarbas Magalhães Resende, Burkhard Bechinger, Rodrigo Moreira Verly

**Affiliations:** 1 Departamento de Química, Faculdade de Ciências Exatas, Universidade Federal dos Vales do Jequitinhonha e Mucuri (UFVJM), Campus JK, Diamantina 39100-000, MG, Brazil; amanda.neves@ufvjm.edu.br (A.N.d.S.); lucio.otavio@ufvjm.edu.br (L.O.N.); kelton.rodrigues@ufvjm.edu.br (K.R.d.S.); 2 Institut de Chimie, Université de Strasbourg, CNRS, UMR7177, 67000 Strasbourg, France; aisenbrey@unistra.fr (C.A.); e.salnikov@gmail.com (E.S.); ahmad.saad@unistra.fr (A.S.); bechinge@unistra.fr (B.B.); 3 Departamento de Química, Instituto de Ciências Exatas, Universidade Federal de Minas Gerais (UFMG), Belo Horizonte 31270-901, MG, Brazil; gabrieleacardoso@ufmg.br (G.d.A.C.); jmr@ufmg.br (J.M.R.); 4 Programa de Pós-Graduação Stricto Sensu em Medicina e Biomedicina, Faculdade de Saúde Santa Casa de Belo Horizonte, Belo Horizonte 30150-221, MG, Brazil; mariaelena@faculdadesantacasabh.edu.br; 5 Institut Universitaire de France, 75005 Paris, France

**Keywords:** antimicrobial activity, *Lycosa erythrognatha* antimicrobial peptide, post-translational modification, peptide–membrane interaction

## Abstract

**Background/Objectives:** This study investigates the structural and biophysical properties of the wild-type antimicrobial peptide LyeTx I, isolated from the venom of the spider *Lycosa erythrognatha*, and its analog LyeTx I-b, designed to enhance antibacterial activity, selectivity, and membrane interactions by the acetylation and increased amphipathicty. **Methods**: To understand the mechanisms behind these enhanced properties, comparative analyses of the structural, topological, biophysical, and thermodynamic aspects of the interactions between each peptide and phospholipid bilayers were evaluated. Both peptides were isotopically labeled with ^2^H_3_-Ala and ^15^N-Leu to facilitate structural studies via NMR spectroscopy. **Results:** Circular dichroism and solid-state NMR analyses revealed that, while both peptides adopt α-helical conformations in membrane mimetic environments, LyeTx I-b exhibits a more amphipathic and extended helical structure, which correlates with its enhanced membrane interaction. The thermodynamic properties of the peptide–membrane interactions were quantitatively evaluated in the presence of phospholipid bilayers using ITC and DSC, highlighting a greater propensity of LyeTx I-b to disrupt lipid vesicles. Calcein release studies reveal that both peptides cause vesicle disruption, although DLS measurements indicate distinct effects on phospholipid vesicle organization. While LyeTx I-b permeabilizes anionic membrane retaining the vesicle integrity, LyeTx I promotes significant vesicle agglutination. Furthermore, DSC and calcein release assays indicate that LyeTx I-b exhibits significantly lower cytotoxicity toward eukaryotic membranes compared to LyeTx I, suggesting greater selectivity for bacterial membranes. **Conclusions**: Our findings provide insights into the structural and functional modifications that enhance the antimicrobial and therapeutic potential of LyeTx I-b, offering valuable guidance for the design of novel peptides targeting resistant bacterial infections and cancer.

## 1. Introduction

Bioactive peptides have been studied as efficient alternatives against pathogenic microorganisms due to their efficiency and specificity [1]. Since the isolation of the LyeTx I peptide from the venom of the *Lycosa erythrognatha* spider, we have been studying the wild-type sequence as well as designed peptide analogs [2]. LyeTx I consists of a potent antimicrobial agent composed of 25 amino acid residues and a natural C-terminal carboxamide (IWLTALKFLGKNLGKHLAKQQLAKL-NH_2_). Its three-dimensional structure in the presence of 400 mM DPC-*d_38_* micelles is characterized by a large extension of *α*-helix conformation and a small random-coil region near the peptide N-terminus [2]. Interestingly, the helix is typified by a non-amphipathic profile, which is quite an unusual feature of natural antimicrobial helical peptides [3]. Other membrane interaction studies revealed the positioning of the random-coil portion into the bilayer interior according to the Trp insertion [2]. Although LyeTx I presents high potential against Gram-positive bacteria *S. aureus* of 3.79 μM, Gram-negative bacteria *E. coli* of 7.81 μM, and yeasts *C. krusei of* 26.30 μM, it is characterized by moderate hemolytic activity. To improve selectivity, modifications within the peptide primary structure were made. The designed analog LyeTx I-b was acetylated at the N-terminus and the His-16 was deleted (CH_3_CO-IWLTALKFLGKNLGKLAKQQLAKL-NH_2_) [4]. LyeTx I-b presented a 10-fold increase in antibacterial activity against Gram-positive bacteria (*Staphylococcus aureus*) with an MIC of 2.85 μM and Gram-negative bacteria (*Escherichia coli*) with an MIC of 0.71 μM against planktonic bacteria. On the other hand, LyeTx I-b exhibited a similar hemolytic capacity against eukaryotic cells comparable to the wide-type sequence [4]. Further biological assays evaluated the anticancer potential and cytotoxic activity against brain tumor cells and nontumoral human cells, including the mechanism of action on glioma cells (U-87 MG), through morphological studies using scanning electron microscopy. LyeTx I-b showed cytotoxicity against brain tumor cancer cells through an effect that involves the induction of necroptosis and, additionally, low hemolytic effect and immunotoxicity [5].

The derivative LyeTx I-b was also tested in a topical formulation (eye drops) to treat resistant bacteria keratitis by intra-stromal injection of 4 μL in female New Zealand white rabbits. The histopathological analysis of the infected eyes demonstrated tissue repair as well as a reduction in the inflammatory process. In addition, LyeTx I-b eye drops were able to treat resistant topical bacterial keratitis in rabbits and proved themselves to be safe for ocular use [6].

Previously, it was observed that the analog LyeTx I-b revealed a higher helical by the deletion of His-16 in organic solvent (tetrafluoroethylene-TFE), ensuring a significant increase in amphipathicity and revealing a stronger peptide–membrane interaction for LyeTx I-b compared to LyeTx I. Also, the pore formation activities of LyeTx I-b and LyeTx I in mimetic membranes. However, the evaluation of the mode of action between both peptides was not observed. Due to significant differences noted in the biological and structural properties of these peptides (Table 1), we decided to investigate the structure–activity relationships further and the evaluation of the mode of action with an increase in the molar ratio. Therefore, this work provides a comparative approach to the structural and thermodynamic aspects of the respective peptide–membrane interactions to describe the effects of both peptides on the phospholipid membrane integrity in higher concentrations, as well as its correlation to changes in antimicrobial activity. A set of biophysical and structural approaches were employed to investigate both peptides in the absence and in the presence of membrane mimetic media.

## 2. Results

### 2.1. Structure and Orientation of the Peptides

#### 2.1.1. Conformational Preferences

**Figure 1:**
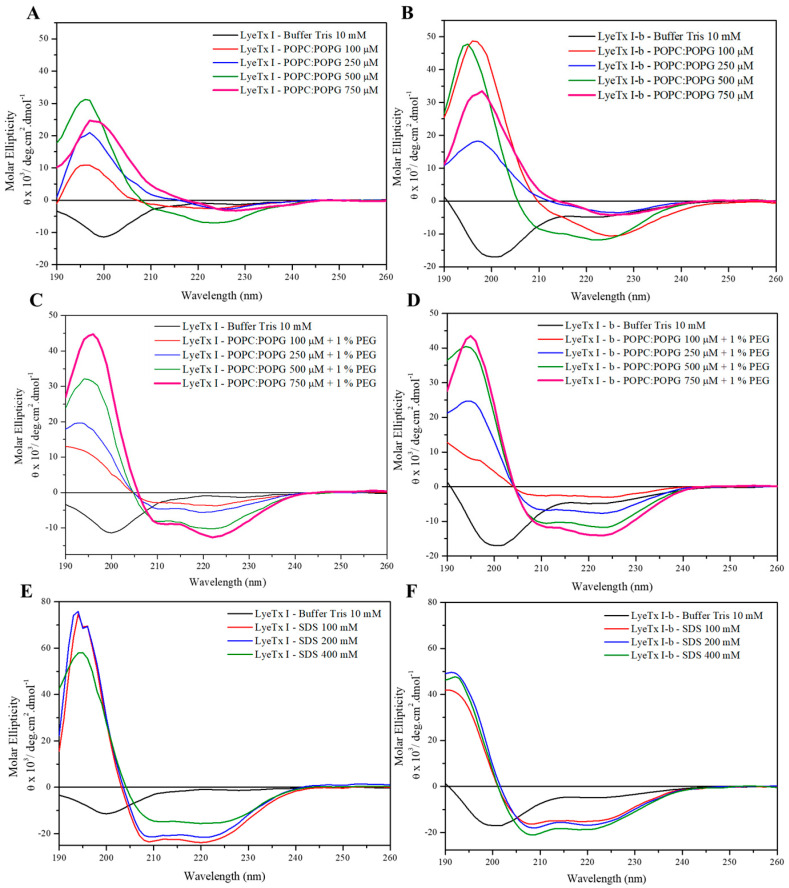
CD spectra of (**A**,**C**,**E**) LyeTx I and (**B**,**D**,**F**) LyeTx I-b in the presence of (**A**,**B**) POPC/POPG (3:1, mol/mol) and (**C**,**D**) POPC/POPG (3:1, mol/mol) + 1% PEGylated POPE (pH 8.0, 20 mM Tris buffer) phospholipid vesicles, as well as in (**E**,**F**) in the presence of SDS micelles.

The conformational preferences of the peptides were investigated by circular dichroism (CD) spectroscopy in the presence of POPC/POPG (3:1, mol/mol) and POPC/POPG (3:1, mol/mol) + 1% PEG phospholipid POPE containing 20 mM Tris buffer (pH 8.0) and 50 μM of each peptide (Figure 1).

The CD spectra indicate that both LyeTx I and LyeTx I-b do not present conformational preferences in the absence of membrane mimetic media (aqueous Tris pH 8.0 buffer—curves in black in Figure 1A–D), as a minimum around 200 nm is observed. However, two minima at ~208 and ~224 nm indicate that both peptides adopt α-helical conformations upon addition of POPC/POPG (3:1, mol/mol) (Figure 1A,B). Interestingly, the molar ellipticity is most pronounced at 500 μM of phospholipids, whereas lower values are observed for higher lipid concentrations (Figure 1A,B). This discontinuity is abolished when 1% PEGylated lipid was added to the POPC/POPG suspensions indicating an aggregation/agglutination effect [8,9,10] in the presence of LyeTx I and lipid concentrations > 500 μm (Figure 1C,D). For LyeTx I-b, an isosbestic point (near 203 nm) was observed.

Experiments in the presence of SDS micelles were performed to check for conformational preferences in a medium suitable for high-resolution solution NMR spectroscopy. Such micelles are amphiphilic in nature with an outer anionic surface, resembling the conditions presented by POPC/POPG LUVs [11]. The spectra of both peptides are characterized by clear a-helices (Figure 1E,F).

#### 2.1.2. Tree-Dimensional NMR Structures

To mimic the negative charge of bacterial membranes, a comparative study of the three-dimensional structures of both peptides was performed using multidimensional solution NMR spectroscopy in the presence of anionic SDS-*d*_25_ micelles. Notably, CD spectroscopy revealed similar helical preferences for both peptides either in the presence of SDS micelles or POPC/POPG LUVs, justifying this choice of medium amenable to solution NMR investigations.

**Figure 2:**
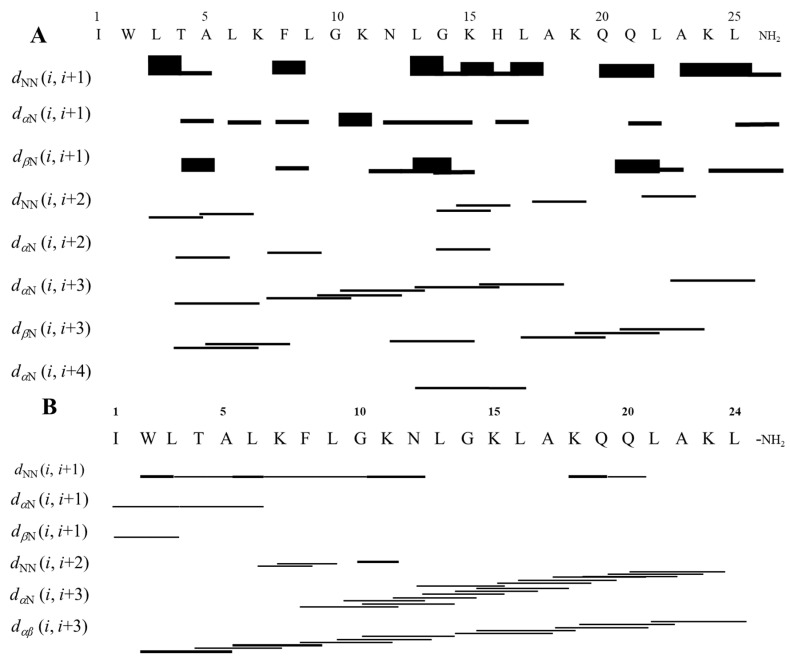
Graphical representation of NOE correlations indicative of helical structures in the NOESY spectra of (**A**) LyeTx I and (**B**) LyeTx I-b in SDS-*d*_25_ micelles.

**Figure 3:**
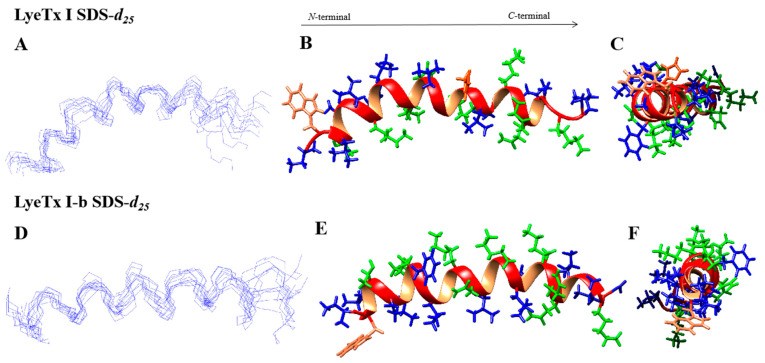
NMR three-dimensional structures of (**A**–**C**) LyeTx I and (**D**–**F**) LyeTx I-b in the presence of 200 mM SDS-*d*_25_ micelles. (**A**,**D**) Superposition of the 10 lowest-energy structures. (**B**,**E**) Horizontal and (**C**,**F**) frontal perspectives of the most stable peptide structure. (**C**) LyeTx I and (**F**) LyeTx I-b structure with the front view of the helix. In (**B**,**C**,**E**,**F**), the hydrophilic residues are shown in green, hydrophobic residues in dark blue, and the Trp residue in orange. The N-terminals are shown in the front in (**E**,**F**) and on the left in (**B**,**E**).

The assignments of intra-residual and inter-residual correlations were performed through joint analysis of homonuclear TOCSY and NOESY spectra. Using the Wüthrich method and the reference values from the Biological Magnetic Resonance Data Bank chemical shift table (available at https://bmrb.io/ref_info/csstats.php, accessed on 16 January 2024), the corresponding spin system for each amino acid residue was identified based on the respective sequential inter-residue correlations [12]. The NOESY spectra contain d_NN_(i, i + 1) correlations involving practically all of the residues within the peptide backbone as well as some d_NN_(i, i + 2) correlations. Additionally, the d_αβ_(i, i + 3) region, also characteristic of helical structures, is observed. In summary, the high occurrence of d_NN_(i, i + 1), d_αN_(i, i + 3), d_αβ_(i, i + 3), and d_αN_(i, i + 4) NOE correlations characteristic of a-helical peptides (Figure 2) is in line with the results obtained from CD spectroscopy. In fact, some d_NN_(i, i + 2) correlations have already been described for LyeTx I-b in aqueous TFE, which were related to the curvature of the peptide helix [2]. Such NOEs were also observed for LyeTx I but to a lesser extent. The NOE intensities and the chemical shift data were converted into geometric restraints, which were used in simulated annealing protocols to obtain the three-dimensional structures of the peptides. As shown in Figure 3, both structures are characterized by a dominant helical segment ranging from Leu-3 to Gln-20 for LyeTx I and from Trp-2 to Lys-23 for LyeTx I-b. The Ramachandran plots (Figure S2) confirm the high stereochemical quality of the 10 lowest-energy structures as >98.0% of the amino acid residues are located in the most favored regions.

The summary of structural statistics of both peptides is shown in (Table 2). LyeTx I shows RMSD values twice as high when compared to the LyeTx I-b, indicating that the N-terminal acetylation and the histidine deletion ensure a higher helical propensity and structural stability for the peptide analog.

#### 2.1.3. Topology of the Peptides in Oriented Membranes

**Figure 4:**
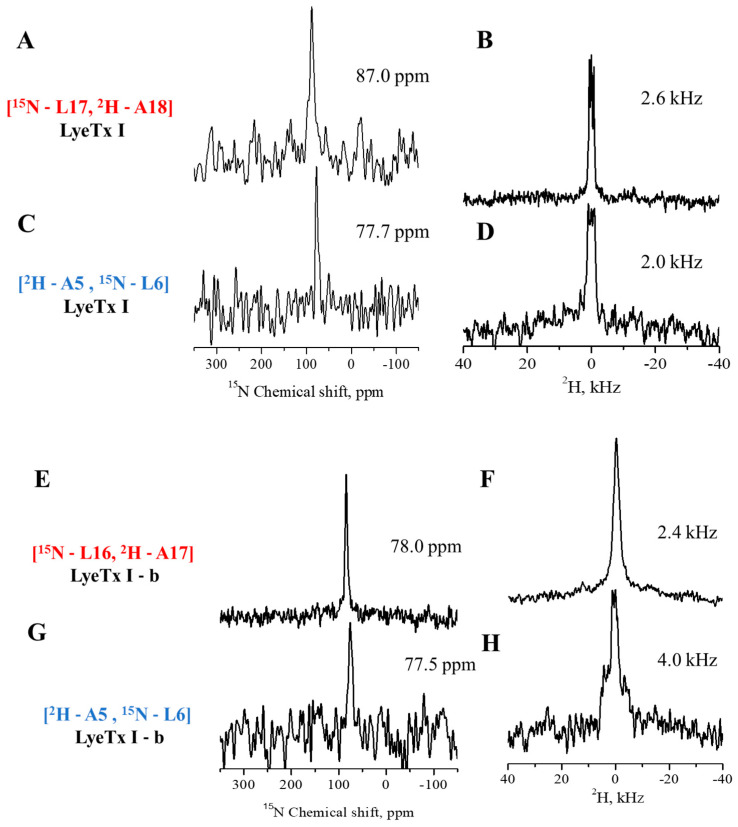
Solid-state NMR spectra of the selectively labeled (**A**–**D**) LyeTx I and (**E**–**H**) LyeTx I-b at 1.0 mol % in uniaxially oriented POPC/POPG (3:1, mol/mol) bilayers. (**A**,**C**,**E**,**G**) Proton-decoupled ^15^N and (**B**,**D**,**F**,**H**) ^2^H solid-state NMR. All spectra were recorded for alignments with the membrane normal parallel to the magnetic field of the spectrometer.

Solid-state nuclear magnetic resonance (ssNMR) experiments were carried out to investigate the topologies of the peptides when interacting with anionic phospholipid membranes. For ssNMR studies, labeling with ^15^N-leucine and ^2^H_3_-alanine was performed with two different labeling schemes for each peptide: LyeTx I labeled at Ala-5/Leu-6 and Leu-17/Ala-18 and LyeTx I-b labeled at Ala-5/Leu-6 and Leu-16/Ala-17. The peptides were reconstituted at a peptide-to-lipid (P/L) ratio of 1 mole% into POPC/POPG (3:1, mol/mol) phospholipid bilayers uniaxially oriented with the membrane normal parallel to the magnetic field of the NMR spectrometer. Proton-decoupled ^31^P (Figure S3), proton-decoupled ^15^N, and ^2^H solid-state NMR spectra were obtained for all samples, as shown in Figure 4.

The ^31^P experiment spectra (Figure S3) are characterized by an intense signal near 30 ppm, characteristic of phospholipids oriented with the main axis parallel to the magnetic field. The ^15^N spectra (Figure 4A,C,E,G) for each labeled site of LyeTx I and LyeTx I-b site were characterized by a single narrow line with chemical shifts below 90 ppm, which is consistent with peptide alignments approximately parallel to the bilayer interface [13].

Whereas (^15^N-Leu-17)-LyeTx I and (^15^N-Leu-16)-LyeTx I-b resonate at 87 ppm and 78 ppm, respectively, the (^15^N-Leu-6)-labeled sites of LyeTx I and LyeTx I-b resonate at very similar chemical shifts of 78 ppm. The ^2^H spectra of LyeTx I are presented in Figure 4B,D,F,H for all ^2^H_3_-Ala-labeled samples. Both labeled ^2^H_3_-Ala18-LyeTx I show a deuterium quadrupolar splitting of 2.6 kHz, while, for ^2^H_3_-Ala-17-LyeTx I-b, the value is 2.4 kHz and 2.4 kHz for ^2^H_3_-Ala-5-LyeTx I-b, whereas ^2^H_3_-Ala5-LyeTx I is 2.0 kHz. However, for spectrum, the determination of the internal rotational polar angle of the helices is not possible. Deuterium quadrupolar splittings < 2.4 kHz are also typical for membrane-associated D-O-H molecules. Therefore, it is possible that the ^2^H splittings of the peptide overlap with the water line but it is also possible that they are absent due to unfortunate motional regimes [14].

### 2.2. Membrane Affinity of the Peptide with Anionic Vesicles

**Figure 5:**
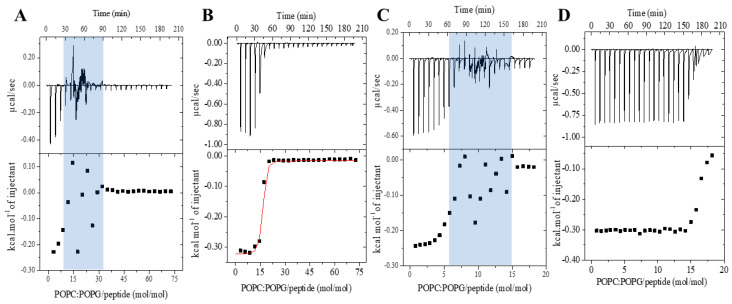
Isothermal titration calorimetric curves obtained from the titration of 20 mM POPC/POPG (3:1, mol/mol) vesicles into Tris-HCl buffer pH 8.0 peptide solutions at 25 °C: (**A**) 50 μM LyeTx I; (**B**) 50 μM LyeTx I-b; (**C**) 100 μM LyeTx I; and (**D**) 100 μM LyeTx I-b. In the panels, the blue shading highlights the molar ratio, while the red line on the curves represents the fitting.

The ITC experiments were conducted to assess the thermodynamic parameters of the peptide–membrane interaction of LyeTx I and LyeTx I-b with POPC/POPG (3:1, mol/mol) phospholipid vesicles. This technique is effective in obtaining the apparent association constant (*K*_app_), ∆*H*°, ∆*S*°, and ∆*G*° parameters by detecting the heat flow during the peptide–membrane interaction [15,16] and previously used to evaluate both LyeTx I and LyeTx I-b with POPC/POPG LUVs (Table S1) [4]. We have further investigated the effect of the peptide concentration and temperature on the membrane interaction of these two peptides. Figure 5 shows the calorimetric titration isotherms acquired for the titration of 20 mM POPC/POPG LUVs into 50 μM and 100 μM peptide solutions at 25 °C. As expected, higher heat flow is noted for LyeTx I-b at both investigated concentrations.

Interestingly, a disturbance in the heat flow is observed from the fourth and eighth injections for the experiment of LyeTx I at 50 μM and 100 μM (Figure 5A), respectively. This phenomenon does not happen at low concentrations at 25 uM but persists within a phospholipid/peptide molar ratio between 7–20 either at 50 μM or at 100 μM of peptide. On the other hand, a continuous decrease in the heat flow is noticed at both peptide concentrations for the system LUVs/LyeTx I-b. A difference in the heat profile between the two peptides is presented as a function of the molar ratio. Notably, both peptides exhibit distinct concentration-dependent modes of interaction. Whereas LyeTx I-b continuously decreases the heat flow of membrane interactions, the wild-type peptide shows a critical peptide–phospholipid ratio at which abrupt changes in membrane interaction occur. This suggests differences in their binding mechanisms or affinities under the tested conditions.

**Figure 6:**
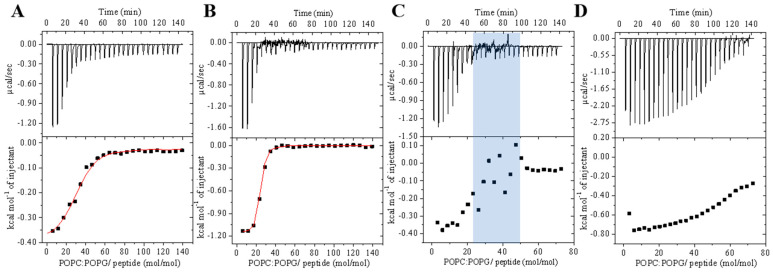
Isothermal calorimetric titration of pH 8.0 Tris-HCl buffered solutions of (**A**,**C**) LyeTx I and (**B**,**D**) LyeTx I-b with 20 mM POPC/POPG (3:1, mol/mol) LUVs: (**A**) 25 μM LyeTx I; (**B**) 25 μM LyeTx I-b; (**C**) 50 μM LyeTx I; and (**D**) 50 μM LyeTx I-b. Experiments were performed at 30 °C. In the panels, the blue shading highlights the molar ratio, while the red line on the curves represents the fitting.

Thermodynamic parameters of the peptide–membrane interaction for each peptide were also evaluated comparatively at a higher temperature, namely 30 °C, at 25 μM and 50 μM of peptide (Figure 6). Once again, a higher heat of interaction is observed for the binding of LyeTx I-b to POPC/POPG vesicles when compared to LyeTx I. Whereas a similar disturbance during the heat flow is once more observed for LyeTx I at 50 μM, such a profile is not observed during the titration of the peptide at 25 μM, even at the higher temperature. Therefore, nonlinear fitting was achieved for each system containing 25 μM of peptide (lower panels of Figure 6A,B), and the obtained thermodynamic parameters are presented in Table 3.

The negative values of Gibbs free energy are consistent with the curve profiles and demonstrate that the process of peptide–membrane interaction takes place spontaneously for both peptides. The thermodynamic parameters further confirm the higher affinity of LyeTx I-b to the predominant anionic POPC/POPG membranes, even at higher temperatures. The *K*_app_ determined for the LyeTx I-b binding is approximately 10^2^ times higher than the value determined for the wild-type sequence.

### 2.3. Effect of the Peptides on the Charge and Size Distribution of Anionic LUVs

The effect of peptide–membrane interaction on the POPC/POPG (3:1, mol/mol) phospholipid LUVs was evaluated by monitoring changes in the hydrodynamic diameter (*D*_h_) and zeta potential (*ζ*) of 500 μM LUVs with increasing concentrations of peptides. The effects of peptide–membrane interactions on the size and surface charge of the LUVs were measured at peptide concentrations ranging from 1 to 80 μM (0.002 to 0.16 peptide–phospholipid molar ration, respectively).

**Figure 7:**
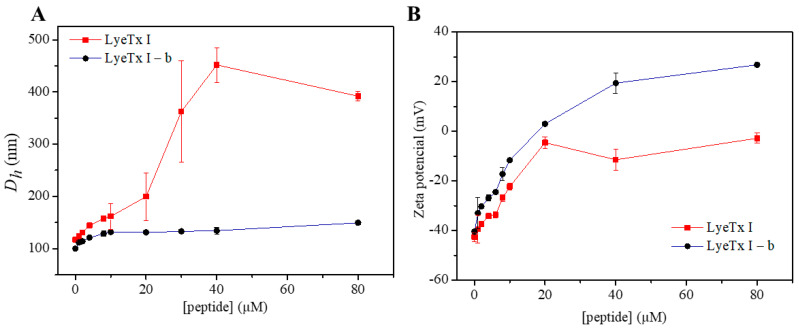
(**A**) Hydrodynamic diameter (*D*_h_) and (**B**) normalized zeta potential (ζ) of POPC/POPG (3:1, mol/mol) LUVs measured at different concentrations of LyeTx I (squares) or LyeTx I-b (circles). LUVs (500 μM) were suspended in 10 mM Tris-HCl buffer (pH 8.0) and 25 °C. Error bars indicate standard deviation from three independent experiments.

The addition of peptide results in increases in both vesicle size and zeta potential (Figure 7). LyeTx I shows a substantially greater increase in the hydrodynamic diameter from 20 μM peptide (*D*_h_ ≅ 200 nm), reaching a maximum at 40 μM LyeTx I (*D*_h_ ≅ 450 nm) with polydispersity index (PDI) values of 0.3, indicating a heterogeneous size distribution [17]. Higher size populations are identified in the graphic distributions (Figure S4), indicating a process of vesicle aggregation/agglutination. On the other hand, the LyeTx I-b peptide causes a regular increase in the LUVs average diameter, reaching a plateau at 5 μM (*D*_h_ ≅ 122 nm). In addition, the polydispersity index (PDI) values remain below 0.3, even at higher peptide concentrations. Consequently, a single-size distribution centered at 125 nm is noted for the LUVs in the presence of 80 μM LyeTx I-b (Figure 7B). Interestingly, the interaction of LyeTx I-b causes a greater increase in the *ζ* potential, especially at peptide concentrations ≥ 40 μM. At the highest concentrations (80 μM), LyeTx I does not achieve charge neutrality and maintains a negative surface. In contrast, LyeTx I-b, despite having a similar charge, is able to neutralize the vesicles in the system, reaching neutrality and making the vesicles positively charged at concentrations above 20 μM.

### 2.4. Comparison of the Effect of Peptides on the Stability of Anionic and Zwitterionic Membranes

#### 2.4.1. Evaluation of Lytic Activity

**Figure 8:**
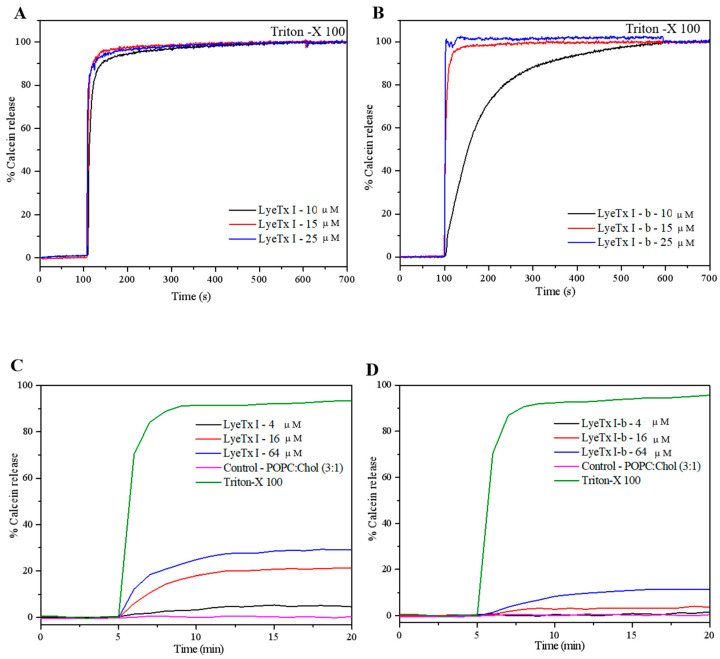
Kinetics of calcein release from (**A**,**B**) POPC/POPG (3:1, mol/mol) and (**C**,**D**) POPC/Chol LUVs induced by different concentrations of (**A**,**C**) LyeTx I and (**B**,**D**) LyeTx I-b.

Dye release studies from calcein-loaded phospholipid vesicles were conducted for LyeTx I and LyeTx I-b in the presence of anionic POPC/POPG (3:1, mol/mol) and of zwitterionic POPC/Chol (3:1, mol/mol) LUVs (Figure 8). The anionic and zwitterionic vesicles were used to mimic bacterial and eukaryotic cells, respectively. Aqueous Triton 100 of 10% *v/v* was added to determine the maximum fluorescence intensity (100% leakage, *I*_max_). LyeTx I causes immediate release of calcein from the POPC/POPG (3:1, mol/mol) vesicles. In contrast, the leakage promoted by LyeTx I-b depends on the peptide concentration, as a slow and gradual dye release from the calcein-loaded anionic LUVs is observed at 10 mM of peptide. The POPC/Chol (3:1, mol/mol) membranes revealed a considerably greater stability in the presence of both peptides, as a maximum release of 20% is observed for LyeTx I at 64 μM, and only 5% of lysis takes places at the lowest tested concentration (4 μM). In its turn, only 10% of dye release is measured for LyeTx I-b at the highest investigation concentration, whereas less than 1% takes place for the peptide at 4 μM.

#### 2.4.2. Effect on the the LUVs Thermotropic Behavior

**Figure 9:**
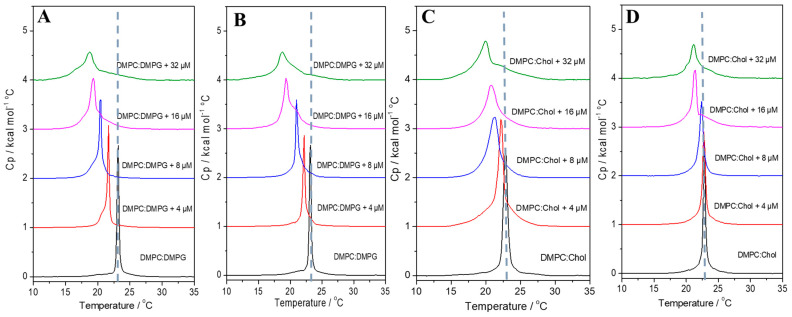
DSC profile of the gel-to-liquid-crystalline phase transition of 3.0 mM (**A**,**B**) DMPC/DMPG and (**C**,**D**) DMPC/Chol in the presence of (**A**,**C**) LyeTx I and (**B**,**D**) LyeTx I-b at different concentrations. Dash grey lines indicate the *T*_m_ of the LUVs in the absence of peptides.

Differential scanning calorimetry (DSC) was used to investigate the thermotropic behavior of DMPC/DMPG (3:1, mol/mol) and DMPC/Chol (3:1, mol/mol) multilamellar vesicles (MLVs) upon the addition of each peptide. The anionic DMPC/DMPG and zwitterionic DMPC/Chol vesicles were used to mimic bacterial and eukaryotic cell membranes, respectively. Additionally, both vesicle systems are more suitable for DSC measurements, as they exhibit main phase transition temperatures (*T_m_*) above 20 °C. Therefore, the effect of peptide–lipid interactions on the *T_m_* and enthalpy changes (Δ*_trans_H*) was evaluated (Table S2). Changes in the thermotropic profile of DMPC/DMPG membranes were identified with the addition of both peptides, resulting in a decrease in the vesicles *T*_m_ in a peptide concentration-dependent manner (Figure 9A,B for LyeTx I and LyeTx I-b, respectively). Both peptides induce a similar shift in *T_m_* of the anionic membranes at all tested concentrations. Additionally, at the highest peptide concentration, the main phase transition peak becomes broader and loses symmetry in the presence of each peptide. There is a clear relationship between Δ*_trans_H* and the increasing concentration of both peptides, causing a concentration-dependent decrease in the main transition enthalpies. However, a lower Δ*_trans_H* (19.0 kJ·mol^−1^) is observed for DMPC/DMPG membranes in the presence of a high concentration of LyeTx I-b compared to the equivalent concentration of LyeTx I (20.2 kJ·mol^−1^).

The peptides apparently present a greater effect on the *T*_m_ of DMPC/Chol vesicles. LyeTx I-b presents a weaker effect on the membrane organization at concentrations below 16 μM, as no significant changes in *T*_m_ are observed (*T*_m_ ≤ 0.42 °C). In its turn, changes in the *T*_m_ promoted by LyeTx I are recognized even at the lowest peptide concentration tested, resulting in a *T*_m_ shift of *T*_m_ ≅ 2.0 °C (Figure 9C,D).

## 3. Discussion

This study provides comprehensive insights into how structural modifications in LyeTx I—specifically, N-terminal acetylation and the deletion of His-16 to produce LyeTx I-b—result in significant enhancements to antimicrobial potency, structural stability, membrane alignment, and interaction depth, culminating in a more selective and potent antimicrobial profile. Antimicrobial assays revealed that LyeTx I-b exhibits substantially higher bactericidal activity than LyeTx I, including against various multidrug-resistant bacterial strains. The mechanism driving this enhanced activity appears to be tied to the optimized peptide–membrane interaction, which is promoted by the increased amphipathicity and stabilized structure of the analog LyeTx I-b [2,4,5].

CD spectroscopy (Figure 1) shows that LyeTx I has a random coil profile in aqueous solution, whereas LyeTx I-b exhibits an additional minimum at ~220 nm, indicating a more stable transition between folded and unfolded states [18,19,20]. This suggests that LyeTx I-b has a more ordered and defined secondary structure, supporting a two-state equilibrium between helix and random coil, unlike LyeTx I [21]. CD spectroscopy and solution structures (Figure 3) confirmed that both peptides adopt an α-helical conformation in membrane mimetic environments. However, LyeTx I-b shows a significantly extended helical structure from residues Trp-2 to Lys-23, compared to the shorter helix in LyeTx I (Leu-3 to Gln-20) [20,21]. Indeed, the deletion of His-16 enhances amphipathicity by aligning the nonpolar residues at the C-terminus with the hydrophobic side chains of the rest of the peptide chain, promoting a well-ordered conformation that interacts parallel to the membrane surface. In addition, acetylation can enhance helix stability by neutralizing the N-terminal charge, allowing deeper insertion of the N-terminal portion and thereby supporting its stronger and more selective membrane interactions. ^15^N ssNMR with selectively labeled residues provided evidence that both LyeTx I and LyeTx I-b align parallel to the lipid bilayer, a characteristic orientation for antimicrobial peptides [22]. However, LyeTx I-b exhibits minimal chemical shift variation between labeled samples (Δδ = 0.5 ppm), indicating a stable membrane-parallel alignment. In contrast, the wild-type LyeTx I shows substantial chemical shift variation (Δδ ≈ 10 ppm) between labeled forms, suggesting less stable alignment. Notably, LyeTx I-b causes a greater increase in ζ potential compared to LyeTx I as a consequence of the higher amount of peptide bound parallel to the membrane surface due to its enhanced amphipathic structure.

Isothermal titration calorimetry (ITC) and differential scanning calorimetry (DSC) reveal that LyeTx I-b inserts more deeply into anionic phospholipid bilayers than LyeTx I due to N-terminal acetylation, which enhances hydrophobic interactions with membrane acetylation [23,24,25]. This increased hydrophobicity allows Trp-2 and the peptide N-terminus of LyeTx I-b to penetrate the bilayer, facilitated by the enhanced amphipathicity. Although both peptides disrupt the *T*_m_ of DMPC/DMPG LUVs, the lower transition enthalpy change (Δ*_trans_H*) for LyeTx I-b (Table S1) indicates that the peptide has a greater impact on the phase transition by disrupting fatty acid chain packing [26,27]. In fact, a higher binding constant (*K*_app_), accompanied by a greater entropic contribution, confirms the enhanced membrane interaction for LyeTx I-b interactions with POPC/POPG vesicles. LyeTx I-b exhibits a more negative Δ*H* than the wild type, while a simultaneous increase in Δ*S* keeps Δ*G* highly favorable. This reflects its optimized design, enhancing binding strength through enthalpic contributions while maintaining entropy gains from desolvation and increased lipid chain disorder.

At 25 °C, LyeTx I-b titration isotherms show greater initial heat flow and a clear inflection point at a 5–15 mol/mol phospholipid/peptide ratio, independently of the peptide concentration. However, LyeTx I exhibits fluctuating heat flow in a similar molar ratio in both used peptide concentrations. This distinction suggests different modes of membrane interaction for each peptide. Interestingly, zeta potential and hydrodynamic diameter measurements (Figure 7) performed at concentrations above and below this molar ratio reflect distinct influences of the peptides on the charge and size distribution of POPC/POPG (3:1) LUVs. Whereas zeta potential and size are similar for both peptides at concentrations below 20 μM, the significant increase in *D_h_* and the broader size distribution of LUVs indicate an aggregation and/or agglutination process at higher concentrations of LyeTx I. In contrast, no significant broadening or additional size distribution of LUVs is observed, even at high concentrations of LyeTx I-b (Figure S4). These findings suggest a controlled permeabilization process caused by LyeTx I-b as opposed to the LyeTx I-induced vesicle agglutination. Therefore, LyeTx I-b enhances its selectivity by reducing the possibility of aggregation-related cytotoxicity, which is often associated with nonselective membrane interactions [10,28,29].

The improved amphipathic character not only explains the higher activity against several bacteria, including multidrug-resistant strains, it also helps to ensure improved selectivity of the LyeTx I-b in comparison with LyeTx I, as previously described [4]. The evidence of the higher membrane insertion in DMPC/Chol of LyeTx I (Figure 9) means that LyeTx I-b causes less disturbance in the packaging of the carbon chain tail of the phospholipid [30]. Consequently, a higher lytic effect of LyeTx I than LyeTx I-b is observed in the calcein releasing from POPC/Chol LUVs lytic activity (Figure 9).

## 4. Material and Methods

### 4.1. Solid-Phase Peptide Synthesis

The peptides LyeTx I and LyeTx I-b were obtained through solid-phase synthesis using the *N*-9-fluorenylmethyloxycarbonyl (Fmoc) strategy on a Rink-amide^®^ resin (0.68 mmol·g^−1^) from Novabiochem^®^ (division of Merck KGaA, Darmstadt, Germany) [31]. Selectively labeled peptides were prepared for solid-state NMR experiments by the incorporation of 3,3,3-^2^H_3_-alanine and ^15^N-leucine Fmoc amino acid derivatives during the respective coupling steps, as indicated in Table 4. The unlabeled peptides were produced manually, whereas the labeled forms were produced using a Millipore 9050 automatic peptide synthesizer (Billerica, MA, USA, division of Merck KGaA, Darmstadt, Germany).

The coupling steps were performed with 2-(1H-benzotriazol-1-yl)-1,1,3,3-tetramethyluronium hexafluorophosphate (HBTU) and *N,N*-diisopropylethylamine (DIPEA) in dimethylformamide (DMF) for 3 h. Fmoc deprotection steps were carried out with 4-methylpiperidine at 25% (*v/v*) in DMF. For the synthesis of LyeTx I-b, the last residue was deprotected and washed to perform the N-terminus acetylation with acetic anhydride (Sigma-Aldrich, St. Louis, MO, USA) at 4% (*v/v*) in DMF for 3 min. For the cleavage step, the respective peptidyl resin was submitted to reaction with a trifluoroacetic acid (TFA)/triisopropylsilane (TIS)/water (95.0:2.5:2.5, *v:v:v*) solution at room temperature during 4 h. The final product was precipitated with cold diethyl ether. Finally, it was resolubilized with 5 mM HCl (hydrochloric acid), frozen with nitrogen, and lyophilized for 48 h.

After the freeze-drying process, the peptides were characterized based on the monoisotopic mass of labeled and unlabeled peptides, as presented in the Supplementary Materials (Figure S1). The theoretical monoisotopic mass of unlabeled LyeTx I is 2830.7 g·mol^−1^ and unlabeled LyeTx I-b is 2735.6 g·mol^−1^, while the labeled ^2^H_3_-ala and ^15^N-leu for LyeTx I is 2835.7 g·mol^−1^ and for LyeTx I-b is 2739.1 g·mol^−1^. The mass spectra of the unlabeled peptides confirm the presence of peaks corresponding to the [M + H]^+^ molecular ions at *m/z* 2832.0 for LyeTx I and *m/z* 2736.8 for LyeTx I-b, and the [M + H]^+^ peaks at *m/z* 2836.7 and *m/z* 2742.42 confirms the presence of the labeled LyeTx I and LyeTx I-b, respectively. Additionally, in the spectra of each peptide, the presence of respective sodium [M + Na]^+^ and potassium [M + K]^+^ ion adducts is identified.

### 4.2. Characterizations and Purification

#### High-Performance Liquid Chromatography (HPLC) and Mass Spectrometry

After freeze-drying, the peptides were purified by reverse-phase HPLC (Gilson, Villiers-le-bel, France) using a preparative C18 column (Luna, C-18-100Å-5 μm, Phenomenex, France) with dimensions of 150 × 30 mm, operated at a flow rate of 20 mL·min^−1^. The fractions were collected automatically and determined by UV absorption of the mobile phase at 214 nm. The gradient of the mobile phase was prepared using solvent A, which contained 10% acetonitrile and 0.1% trifluoroacetic acid (TFA) in Milli-Q water, and solvent B, consisting of 100% acetonitrile with 0.1% TFA. For each peptide, the gradient of acetonitrile began at 30% and was increased up to 70%.

The identity and purity of the collected fractions were determined by MALDI-TOF mass spectrometry (Autoflex^®^ Bruker Daltonics, Bremen, Germany). The aqueous solution of acetonitrile from the main peak, containing 0.1% TFA, along with 10 μL of a saturated solution of α-cyano-4-hydroxycinnamic acid (CHCA) matrix, was applied to an MTP AnchorChip^®^ 400/384 plate (Bruker Daltonics^®^, Bremen, Germany) and dried at room temperature. Once the monoisotopic mass of the desired peptide was identified, the respective fraction was dried under vacuum with a rotary evaporator to form a film. The dried and pure film was dissolved three times in 5 mM hydrochloric acid (HCl) and transferred to clean, weighed tubes, followed by lyophilization to ensure the exchange of the TFA counter-ions. Peptide quantification relied on the mass obtained after lyophilization and reweighing. Hereafter, the peptides were stored at −20 °C until usage.

### 4.3. Preparation of Large Unilamellar Vesicles (LUVs)

Large unilamellar vesicles (LUVs) were produced using a mixture of 1-palmitoyl-2-oleoyl-sn-glycero-3-phospho-(1-glycerol) (POPG) and 1-palmitoyl-2-oleoyl-sn-glycero-3-phosphocholine (POPC) in a 3:1 molar ratio. The lipids were placed into a glass tube, dissolved in chloroform at ambient temperature, and then subjected to a stream of nitrogen gas to evaporate the solvent, resulting in a lipid film. The lipid film was subsequently dried under vacuum in a lyophilizer for a minimum of 24 h. Thereafter, the lipid film was rehydrated with an aqueous buffer (100 mM Tris-HCl, pH 8). The resulting multilamellar vesicles (MLVs) were subjected to eight freeze/thaw cycles using liquid nitrogen, a water bath at 35 °C, and vortex mixing. The MLVs were extruded in a 10 mL Avanti Polar Lipids Inc.^®^ mini extruder (Alabaster, AL, USA) using polycarbonate membranes with 100 nm pore size (Whatman Nuclepore, Sigma-Aldrich, ) at 25 °C to obtain LUVs of 100 nm. This LUVs preparation was used for experiments of isothermal titration calorimetry (ITC), zeta potential/dynamic light scattering (DLS), and circular dichroism (CD) experiments. To investigate the effect of aggregation, phospholipid vesicles containing 1% of PEGylated phospholipid POPE were also prepared.

For the calcein release experiments, the preparation of LUVs followed the same principle but, in this case, it was necessary to incorporate the fluorophore into the solution. For this proposal, 2.0 mL of a 50 mM calcein diluted in 100 mM Tris-HCl buffer (50 mM NaCl, pH 7.4) was added during the process of multilamellar vesicles (MLVs) preparation. To prepare the fluorophore buffered solution, 545 mg of calcein was dissolved in 2.5 mL of 1 M NaOH; then, 1.25 mL of 100 mM Tris-HCl buffer (50 mM NaCl, pH 7.4) and 2.5 mL of Milli-Q water were added. The pH was adjusted to 7.4 with NaOH under stirring to prevent precipitation [32]. MLVs encapsulated with calcein were submitted to eight freeze/thaw cycles using liquid nitrogen and a water bath at 35 °C and, finally, extruded in a 10 mL stainless steel extruder (Lipex Biomembranes Inc., Vancouver, BC, Canada) to obtain 100 nm LUVs. To separate dye-containing vesicles from non-entrapped calcein, a size exclusion chromatographic column (Sephadex G50 Fine, 10 × 150 mm) was used, eluted with 10 mM Tris-HCl buffer. The total lipid concentration of the extruded LUVs was determined as described by Avanti Polar Lipids [33].

### 4.4. Conformational and Topological Studies

#### 4.4.1. Circular Dichroism Spectroscopy

CD spectroscopy was used to evaluate the secondary structure profiles of LyeTx I and LyeTx I-b in the presence of biomimetic media. The spectra were carried out in the presence of SDS micelles as well as in the presence of 100 nm unilamellar vesicles made of POPC/POPG (3:1, mol/mol) or POPC/POPG (3:1, mol/mol) + 1% PEG-POPE at different concentrations, both suspended in 10 mM Tris-HCl Buffer, pH 8.0. Spectra of both peptides were also acquired in the absence of biomimetic environments, with peptide solubilized only in 10 mM Tris-HCl Buffer. The experiments were recorded on a JASCO^®^ J-810 spectropolarimeter (Tokyo, Japan) coupled to a Jasco Peltier temperature control system—PFD425S. A quartz cuvette with a 1.0 mm optical path was used and the temperature was maintained at 25 °C. For each experiment, five scans were performed with a spectral window from 190 to 260 nm. Data processing and deconvolution calculations to obtain structural contents were processed using Spectra Analysis^®^ 9.3.0 and CDPro^®^ software version 3.1 [31].

#### 4.4.2. Solution NMR Spectroscopy 

Nuclear magnetic resonance (NMR) experiments were carried out in solution for a 600 μL sample containing 4.0 mM LyeTx I-b in the presence of 200 mM SDS-*d*_25_ micelles containing 5% (*v/v*) D_2_O and, for 2 mM LyeTx I, in the presence of 200 mM SDS-*d*_25_ micelles containing 10% (*v/v*) D_2_O. 2,2-dimethyl-2-silapentane sulfonate (DSS) at 1.0 mM was used as an internal reference for both samples. Solution NMR spectra were obtained on a Bruker^®^ 600 MHz Avance Neo spectrometer (Bruker Daltonics Inc., Billerica, MA, USA). Two-dimensional homonuclear TOCSY and NOESY experiments were carried out [34] to determine the chemical shifts of the hydrogen nuclei. ^1^H-^13^C HSQC was recorded to access ^13^C data. The TOCSY experiments were acquired using the DIPSI-2 scheme [35] using a spin-lock time of 120 ms. NOESY spectra were acquired using different mixing times (60, 100, 120, and 150 ms) to check for spin diffusion. The parameters used for the TOCSY experiments for LyeTx I-b were a spectral width of 7500 Hz and 256 *t*_1_ increments with 104 transients of 4096 points; for LyeTx I, a spectral width of 6009.6 Hz (12 ppm) with 96 transients of 4096 points was used. The parameters used for NOESY experiments of LyeTx I-b were a spectral width of F2 7500 Hz and 512 *t*_1_ increments with 112 transients of 4096 points; for LyeTx I, they were a spectral width of 6009, 15 Hz (12 ppm), and 512 *t*_1_ increments with 114 transients of 4096 points. The ^1^H-^13^C spectral data were acquired in a phase-sensitive fashion such that CH and CH_3_ correlations show positive phase, whereas CH_2_ correlations have negative phase [36]. Regarding the acquisition parameters for the LyeTx I-b sample, F2 spectral widths of 7500 Hz and F1 of 31,444 Hz were used, respectively, in 256 *t*_1_ increments with 150 transients of 4096 points and, for LyeTx I, F2 spectral widths of 6009 Hz and F1 of 11,319 Hz were used, respectively, in increments with 150 transients of 4096 points. The two-dimensional spectra were processed using the NMRPipe^®^ version 8.9 computational package [37].

#### 4.4.3. NMR Data Analysis and Structure Calculations

The NMR contour maps were manually assigned using the NMRViewJ^®^ software (version 9.2.0-b24) [38]. Chemical shifts of all spin systems were referenced to the Protein NMR and Biological Magnetic Resonance Data Bank (http://bmrb.io/ref_info/csstats.php, accessed on 16 January 2024). NOE intensities recorded at 120 ms mixing times were converted into semi-quantitative distance restrains by using calibrations by Hybert et al. [39]. The resulting upper distance limits were set at 2.8 Å, 3.4 Å, and 5.0 Å for strong, medium, and weak NOEs, respectively. Angular restraints were determined based on the analysis of C_α_, H_α_, H_β_, C_β_, and H_N_ chemical shifts using the TALOS+^®^ program [40]. Structure calculations were performed using the XPLOR-NIH^®^ software (version 2.27) [41,42]. A total of 100 structures were generated using a simulated annealing protocol starting with an extended conformation. The calculations were performed with an initial temperature of 1000 K, followed by 14,000 steps at high temperatures and 6000 steps during each cooling stage. Cooling was performed in 50 K increments until the temperature reached 100 K. The 20 lowest-energy structures were refined by sa_new.inp protocol. The stereochemical quality of the refined structures was assessed using PROCHECK-NMR version 1.5 [43]. MOLMOL software version 1.0. 7 was used for visualization, structural analysis, and adjustments of the 3D models [43].

#### 4.4.4. Solid-State NMR Spectroscopy 

To prepare the peptide–lipid samples, a solution with a final peptide-to-lipid (P/L) molar ratio of 1/99 mol/mol was prepared. A mixture of labeled peptide and POPC/POPG (3:1, mol/mol) phospholipids was solubilized in a mixture of chloroform/methanol (1:1). After homogenizing the sample, the solvent was evaporated under a stream of nitrogen to reduce its total volume to about 400 mL. Once a viscous sample was deposited onto 22 ultrathin glass plates of dimensions 8 × 18 mm^2^ (Marienfeld, Lauda-Königshofen, Baden-Württemberg, Germany) and dried under a high vacuum to completely remove the solvent. The solvent-free lipid films were transferred to a hydration chamber equilibrated at 93% relative humidity with ^2^H-depleted water. After hydration, the plates were stacked on top of each other; then, the obtained stack was wrapped with Teflon^®^ tape and sealed with a plastic wrapping [44,45].

The experiments were carried out on a Bruker Avance 300 MHz (Bruker BioSpin GmbH, Rheinstetten, Baden-Württemberg, Germany) wide-bore spectrometer using a commercial triple resonance probe for solid-state NMR. Proton-decoupled ^31^P experiments were performed at 121.5 MHz using a Hahn-echo pulse sequence [46] with a 90° pulse width of 8 μs, spectral width of 100 kHz, echo delay of 100 μs, a recycling delay of 3 s, and an acquisition time of 7.7 ms. Exponential apodization of 100 Hz was applied prior to Fourier transformation. The spectra were referenced with a standard solution of 85% H_3_PO_4_ at 0 ppm.

Proton-decoupled ^15^N experiments were acquired at 30.4 MHz on a Bruker Avance 300 MHz (Bruker BioSpin GmbH, Rheinstetten, Baden-Württemberg, Germany) wide-bore spectrometer using a cross-polarization pulse sequence [46]. The following parameters were used: CP contact time of 400 μs, 90° pulse length of 8 μs, recycle delay of 3.5 s, 45 k acquisitions, and a 25 kHz spectral window. Exponential apodization of 50 Hz was applied prior to Fourier transformation. A sample of ^15^NH_4_Cl (39.3 ppm) was used as an external reference.

^2^H NMR experiments were carried out at 76.76 MHz using a quadrupole echo pulse sequence [46] on a Bruker Avance DSX-500 MHz (Bruker BioSpin GmbH, Rheinstetten, Baden-Württemberg, Germany) wide-bore spectrometer using a commercial static triple resonance probe. The following acquisition parameters were used: spectral window of 1000 kHz, acquisition time of 8.2 ms, 90° pulse length of 4.65 μs, recycle delay of 300 ms, echo delay of 100 μs, 16 k time domain, and 450 k scans. Exponential apodization of 300 Hz was applied prior to Fourier transformation. The spectra were calibrated in relation to ^2^H_2_O (0 Hz).

### 4.5. Interaction with Membrane

#### 4.5.1. Isothermal Titration Calorimetry (ITC)

ITC was performed at 25 °C and 30 °C for both LyeTx I and LyeTx I-b with interactions of POPG/POPC (3:1, mol/mol) vesicles. All experiments were recorded in a Malvern^®^ VP-ITC microcalorimeter (Malvern Panalytical Ltd., Malvern, UK). In this experiment, a procedure similar to Munhoz and collaborators (2021) [47] was used. For each injection, a total of 25 successive injections of 5.0 μL of a 20 mM vesicle solution were introduced into a cell containing peptide solutions at concentrations of 25, 50, and 100 μM. The data collection and isothermal treatments for each analysis were executed using Microcal Origin 7.0 software specifically designed for ITC. To determine the partial molar enthalpy of complexation (=*dQ*/*d*[*X*]) at constant pressure, nonlinear fitting was performed by using the model of one-site binding based on the Wiseman Isotherm [22] (Equation (1)) to obtain:

(1) $$\bar{{∆}_{comp}H}={\left(\frac{dQ}{d{\left\lceil X\right\rceil }_{tot}}\right)}_{P}={∆}_{int}H{}^{\circ}V_{0}\left[\frac{1}{2}+\frac{1-X_{R}-r}{\sqrt[{2}]{{\left(1+X_{R}-r\right)}^{2}-4X}}\right]$$

#### 4.5.2. Dynamic Light Scattering and Zeta Potential

The effect of each peptide on the size and charge of 100 nm POPC/POPG (3:1, mol/mol) phospholipid vesicles (LUVs) was evaluated by dynamic light scattering (DLS) and zeta potential (ζ) measured on a Zetasizer nano ZS BI-900 particle analyzer from Malvern (Malvern, Worcestershire, UK), equipped with a 10 mW Ele-Ne laser, λ 633 nm. Experiments were performed at 25 °C by titrating each peptide to 8.0 mM in 500 μM POPC/POPG LUVs, both suspended in 10.0 mM Tris-HCl buffer, pH 8.0. After each injection, a 20 min interval was allowed for the system to stabilize before measuring the hydrodynamic diameter (*D*_h_) and the zeta potential (ζ).

#### 4.5.3. Differential Scanning Calorimetry (DSC)

The peptides were analyzed based on the phase transition profiles of 3 mM 1,2-dimirystoyl-sn-glycero-3-phosphocholine/cholesterol (DMPC/Chol) and 1,2-dimyristoyl-sn-glycero-3-phosphocholine/1,2-dimyristoyl-sn-glycero-3-phosphoglycerol DMPC/DMPG). The experiments conducted in the absence and the presence of LyeTx I or LyeTx I-b at 4, 8, 16, and 32 μM were examined using a VP-DSC^®^ microcalorimeter (Malvern^®^ Instruments, Malvern, Worcestershire, UK). All LUVs and peptide–LUVs mixtures were freshly prepared prior to the experiments. Degassed samples of LUVs and peptide–LUVs were measured against 10 mM Tris-HCl buffer (pH 8.0) containing 50 mM NaCl in the reference cell. Control experiments with buffer in both cells were conducted to allow for blank corrections. Each sample underwent three consecutive heating scans over a temperature range of 10–35 °C at a heating rate of 1.0 °C/min. Data analysis, including blank subtraction, calculation of the transition temperature (*T_m_*—gel to liquid crystalline), and determination of the phase transition enthalpy (Δ*_trans_H*), was performed using Microcal Origin^®^ 9.1 DSC software (GE HealthCare-Microcal^®^, Northampton, MA, USA). A linear baseline was used to integrate the areas under the DSC curves for the calculation of Δ*_trans_H*.

#### 4.5.4. Fluorescence Spectroscopy

##### Calcein Release Assays

The membrane permeation capabilities of LyeTx I and LyeTx I-b were evaluated at 25 °C by measuring calcein efflux from LUVs using a Cary Eclipse Fluorescence Spectrophotometer (Agilent, Palo Alto, CA, USA) with excitation wavelength of 490 nm and emission wavelength of 515 nm. A total of 250 μL of POPC/POPG (3:1, mol/mol) calcein-loaded LUVs were added to a fluorescence cuvette containing Tris-HCl buffer (pH 8). The increase in calcein fluorescence as a function of time was measured continuously after adding different concentrations of the peptide (10, 15, and 25 μM). After 600 s, 10 μL of 10% (*v/v*) Triton X-100 was added to the cuvette to obtain complete vesicle leakage and maximum fluorescence intensity. The percentage of calcein release was calculated according to Equation (2):

(2) $$\%Leakage=\frac{I_{0}-I_{t}}{I_{0}-I_{max}}\times 100$$

where *I*_0_ is the fluorescence prior to the addition of peptide, *I*_t_ is the measured time-dependent fluorescence after peptide addition, and *I_max_* the fluorescence after addition Triton X-100.

## 5. Conclusions

In conclusion, this study provides a comprehensive analysis of the structural, biophysical, and thermodynamic properties of LyeTx I and its modified form, LyeTx I-b. The higher antimicrobial activity of LyeTx I-b is closely correlated with its improved structural amphipathicity, membrane alignment, and insertion depth, factors which cumulatively contribute to its stronger membrane interactions. From a structural perspective, stabilization at the N-terminus and increased amphipathicity were observed. These properties are common features of antimicrobial peptides, aligning with the greater antimicrobial potential of LyeTx I-b. LyeTx I-b has a binding constant approximately 100 times higher than that of LyeTx I, along with a more favorable entropic contribution to binding, indicating that LyeTx I-b’s interaction with bacterial membranes is not only spontaneous but also highly energetically efficient. This enhanced binding affinity stems from the amphipathic profile of the analog, which promotes stronger hydrophobic interactions with bacterial membranes while avoiding destabilization of mammalian cell membranes. Additionally, zeta potential measurements indicated that LyeTx I-b reaches charge neutrality on bacterial membranes at lower concentrations than LyeTx I, suggesting an improved ability to neutralize bacterial surfaces, a critical factor in the selectivity and potency of antimicrobial peptides.

These biophysical properties reflect a classic antimicrobial mechanism, where optimized amphipathic and helical structures enable the peptide to act preferentially on bacterial rather than mammalian membranes, as seen in selective antimicrobial peptides. Together, the thermodynamic, structural, and biophysical data confirm that changes in the LyeTx I structure significantly enhance its selectivity and potency, establishing it as a promising candidate for antimicrobial applications.

## Figures and Tables

**Table 1:** Physicochemical characteristics of the peptides.

Property	LyeTx I	LyeTx I-b
Sequence	IWLTALKFLGKNLGKHLAKQQLAKL-NH_2_	CH_3_CO-IWLTALKFLGKNLGKLAKQQLAKL-NH_2_
Net charge (pH 7)	+5	+4
Length (residues)	25	24
Hydrophobic residues	I, W, L, A, L, F, L, L, L, A, L, A, L (13 total)	I, W, L, A, L, F, L, L, L, A, L, A, L (13 total)
Hydrophobicity (*H*)	0.523 *	0.539 *
Hydrophobic moment (*μ*)	0.198 *	0.426 *
Molecular weight (Da)	2831.7	2735.6
N-terminal modification	None	Acetylation (-CH_3_CO)
C-terminal modification	Amidation (-NH_2_)	Amidation (-NH_2_)

* The data were calculated by https://heliquest.ipmc.cnrs.fr/cgi-bin/ComputParams.py (accessed on 20 Jan 2024) [7].

**Table 2:** NMR and refinement statistics for structures of 2.0 mM LyeTx I and 4.0 mM LyeTx I-b in the presence of 200 mM SDS-*d*_25_ at 25 °C.

	LyeTx I	LyeTx I-b
NMR distance and dihedral constraints	310	238
Distance constraints		
Total NOE		
Intra-residue	195	137
Inter-residue		
Sequential (|*i* − *j*| = 1)	63	28
Medium-range (|*i* − *j*| < 4)	52	33
Total dihedral angle restraints	25	40
Structure statistics		
Ramachandran analysis		
Residues in most favored regions	100%	99.0%
Residues in additional allowed regions	0.0%	1.0%
Residues in generously allowed regions	0.0%	0.0%
Residues in disallowed regions	0.0%	0.0%
Average pairwise r.m.s. deviation ** (Å)		
Residue 1 to 12—Backbone	0.93	0.56 Å
Residue 12 to 25—Backbone	0.98	0.88 Å

** Pairwise r.m.s. deviation was calculated among 10 refined structures.

**Table 3:** Thermodynamic parameters obtained from the titration of LyeTx I and LyeTx I-b (25 μM) with POPC/POPG LUVs (20 mM).

Peptide	*K*_app_ (L·mol^−1^)	∆*G*° (cal·mol^−1^)	∆*H*° (cal·mol^−1^)	*T*∆*S*° (cal·mol^−1^)
LyeTx I ^a^	1.8 × 10^4^ ± 1.6 × 10^3^	−5902	−410 ± 20	18.1 ± 0.9
LyeTx I-b ^a^	1.0 × 10^6^ ± 1.7 × 10^5^	−8308	−1150 ± 50	23.6 ± 1.2
LyeTx I-b ^b^	1.2 × 10^6^ ± 1.5 × 10^5^	−6881	−721 ± 250	25.5 ± 7.5

^a^ Fitting values from experiments at 30 °C and ^b^ fitting values from experiments at 25 °C.

**Table 4:** Primary structures of LyeTx I and LyeTx I-b peptides prepared for these investigations.

**Peptides**	**Characteristics**	**Sequences**
LyeTx I	Unlabeled	IWLTALKFLGKNLGKHLAKQQLAKL-NH_2_
	Labeled [^15^N—L17, ^2^H—A18]	IWLTALKFLGKNLGKHL**A**KQQLAKL-NH_2_
	Labeled [^2^H—A5, ^15^N—L6]	IWLT**A**LKFLGKNLGKHLAKQQLAKL-NH_2_
LyeTx I-b	Unlabeled	CH_3_CO-IWLTALKFLGKNLGKLAKQQLAKL-NH_2_
	Labeled [^15^N—L16, ^2^H—A17]	CH_3_CO-IWLTALKFLGKNLGKL**A**KQQLAKL-NH_2_
	Labeled [^2^H—A5, ^15^N—L6]	CH_3_CO-IWLT**A**LKFLGKNLGKLAKQQLAKL-NH_2_

^15^N-Leu-labeled residues are underlined, whereas the 3,3,3-^2^H_3_-Ala-labeled residues are presented in bold.

## Data Availability

Data are contained within the article and Supplementary Materials.

## Supplementary Materials

The following supporting information can be downloaded at: https://www.mdpi.com/article/10.3390/antibiotics14010066/s1, Figure S1: Graphic of raw samples of peptides isotopically labeled by the MALDI-ToF/ToF method; Figure S2: Ramachandran plot for the 10 lowest-energy structures of (**A**) LyeTx I and (**B**) LyeTx I-b in the presence of SDS-d_25_. Figure S3: Solid-state NMR spectra of proton-decoupled ^31^P of the selectively labeled (**A**,**C**) LyeTx I and (**B**,**D**) LyeTx I-b at 1.0 mol % in uniaxially oriented POPC/POPG (3:1, mol/mol) bilayers; Table S1: Thermodynamic parameters obtained after one binding experiment, nonlinear adjustment for 25 μM LyeTx I or LyeTx I-b interaction with 20 mM POPC/POPG (3:1, mol/mol) LUV at 25 °C; Figure S4: Polydispersity Index (PDI) histograms; Table S2: Transition temperature (Tm) and transition enthalpy change (ΔtransH) values for the DMPC/DMPG (3:1) and DMPC/Chol (3:1) LUVs in the presence of both peptides. The results are presented as a function of peptide concentration.
