# Supplementary material for: Comparative Structural and Biophysical Investigation of Lycosa erythrognatha Toxin I (LyeTx I) and Its Analog LyeTx I-b
Source: Antibiotics (Basel). 2025 Jan 10;14(1):66. doi: 10.3390/antibiotics14010066 (PMC11762800; doi:10.3390/antibiotics14010066)
Supplement: Supplementary file 1 [file antibiotics-14-00066-s001.zip › antibiotics-3318949-supplementary.pdf]

antibiotics

Article

# Comparative Structural and Biophysical Investigation of *Lycosa erythrognatha* Toxin I (LyeTx I) and its Analog LyeTx I-b

**Amanda Neves de Souza [1,2], Gabriele de Azevedo Cardoso [3], Lúcio Otávio Nunes [1], Christopher Aisenbrey [2], Evgeniy Salnikov [2], Kelton Rodrigues de Souza [1,2], Ahmad Saad [2], Maria Elena de Lima [5], Jarbas Magalhães Resende [3], Burkhard Bechinger [2,4] and Rodrigo Moreira Verly [1,]***

1 Departamento de Química, FACET, Universidade Federal dos Vales do Jequitinhonha e Mucuri (UFVJM)—Campus JK, Diamantina, MG, Brazil; amanda.neves@ufvjm.edu.br (A.N.S)
2 Institut de Chimie, Université de Strasbourg/CNRS, UMR7177, Strasbourg, France; esalnikov@unistra.fr (E.S); aisenbrey@unistra.fr (C.A); kelton.rodrigues@ufvjm.edu.br (K.R.S)
3 Departamento de Química, Instituto de Ciências Exatas, Universidade Federal de Minas Gerais (UFMG), Belo Horizonte, MG, Brazil; gabrieleacardoso@ufmg.br (G.A.C); jmr@ufmg.br (J.M.R.)
4 Institut Universitaire de France, Paris, France; bechinge@unistra.fr (B.B)
5 Faculdade de Saúde Santa Casa de Belo Horizonte, Programa de Pós-Graduação Stricto Sensu em Medicina e Biomedicina, Faculdade Santa Casa de Belo Horizonte, Belo Horizonte, MG, Brazil; mariaelena@faculdadesantacasabh.edu.br (M.E.L.)
* Correspondence: verly.rodrigo@ufvjm.edu.br (R.M.V.)

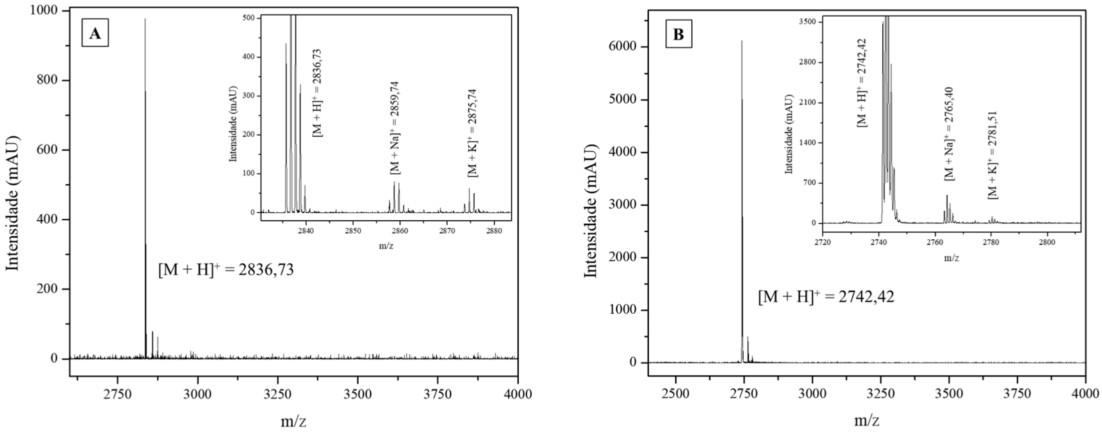


**Figure S1:** Graphic of raw samples of peptides isotopically labeled by the MALDI-ToF/ToF method. (A) LyeTx I and (B) LyeTx I-b.

 https://doi.org/10.3390/antibiotics14010066

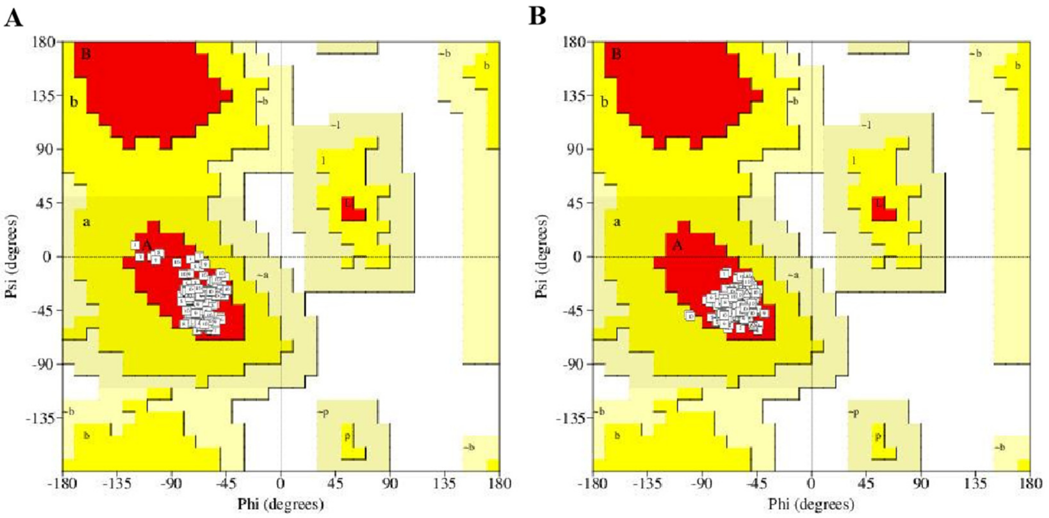


**Figure S2:** Ramachandran plot for the 10 lowest-energy structures of (A) LyeTx I and (B) LyeTx I-b in the presence of SDS-$d_{25}$.

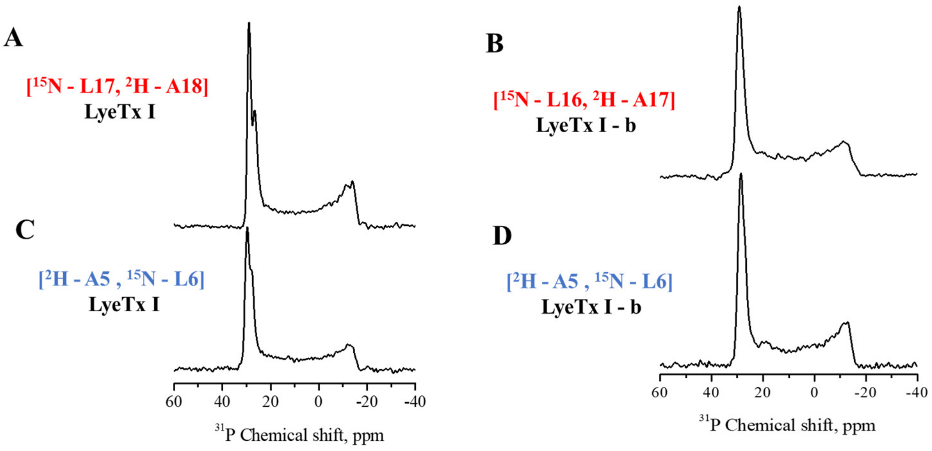


**Figure S3:** Solid-state NMR spectra of Proton-decoupled $^{31}P$ of the selectively labeled (A and C) LyeTx I and (B and D) LyeTx I-b at 1.0 mol % in uniaxially oriented POPC:POPG (3:1, mol:mol) bilayers.

**Table S1:** Thermodynamic parameters obtained after one binding experiment, non-linear adjustment for 25 µM LyeTx I or LyeTx I-b interaction with 20 mM POPC:POPG (3:1, mol:mol) LUV at 25°C.*

| Peptides | $K_{app}$ (L.mol$^{-1}$) | $\Delta G°$ (cal.mol$^{-1}$) | $\Delta H°$ (cal.mol$^{-1}$) | $\Delta S°$ (cal.mol$^{-1}$) |
|---|---|---|---|---|
| LyeTx I | 3.090 ± 150 | -6100 | -210 ± 05 | 19.8 |
| LyeTx I-b | 7.270 ± 350 | -14200 | -7600 ± 50 | 22.0 |

* P. V. M. Reis et al., "LyeTxI-b, a Synthetic Peptide Derived From Lycosa erythrognatha Spider Venom, Shows Potent Antibiotic Activity in Vitro and in Vivo," Front Microbiol, vol. 9, Apr. 2018, doi: 10.3389/fmicb.2018.00667.

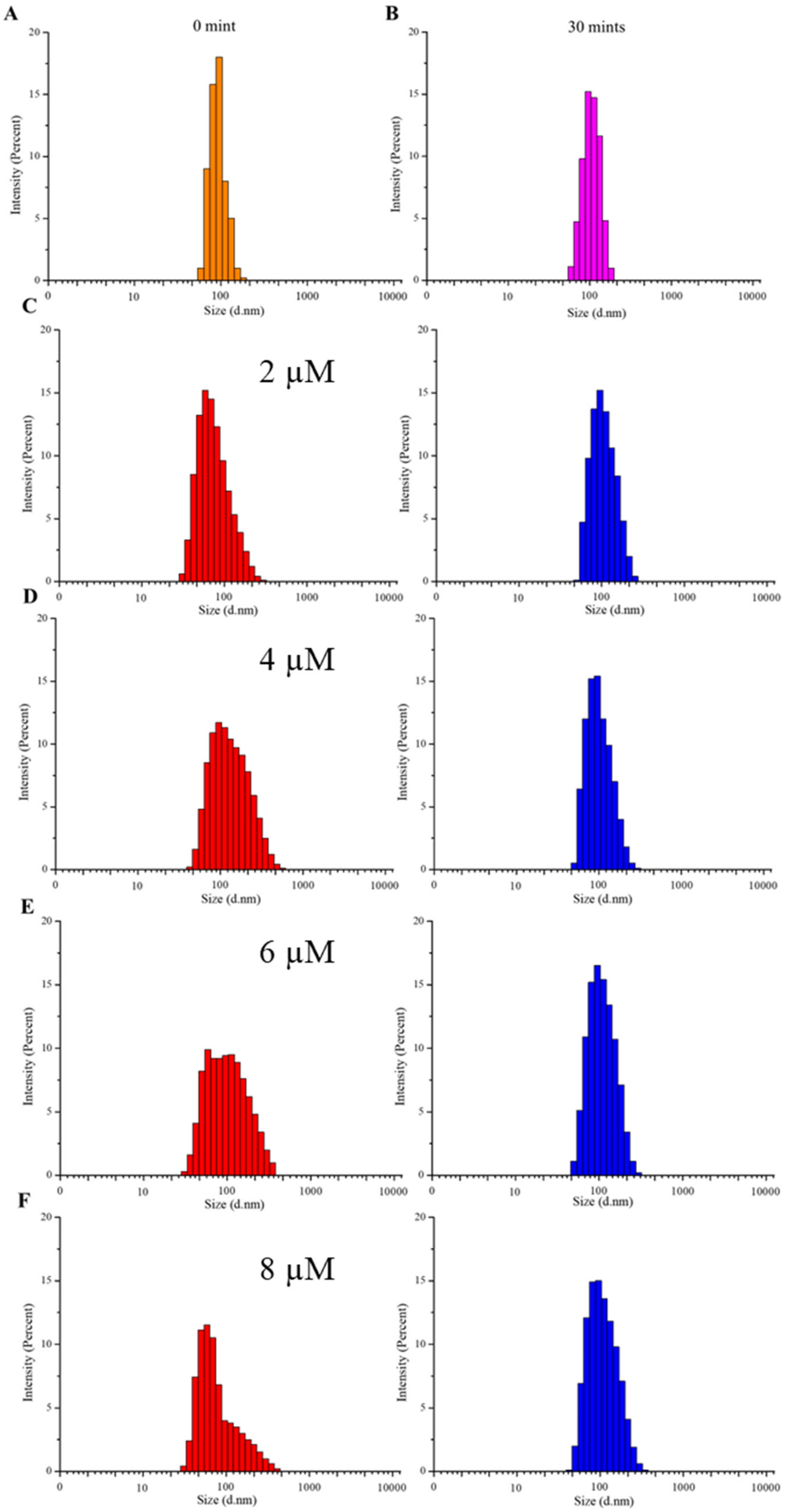
A
0 mint
B
30 mints
C
2 μM
D
4 μM
E
6 μM
F
8 μM
Intensity (Percent)
Size (d.nm)

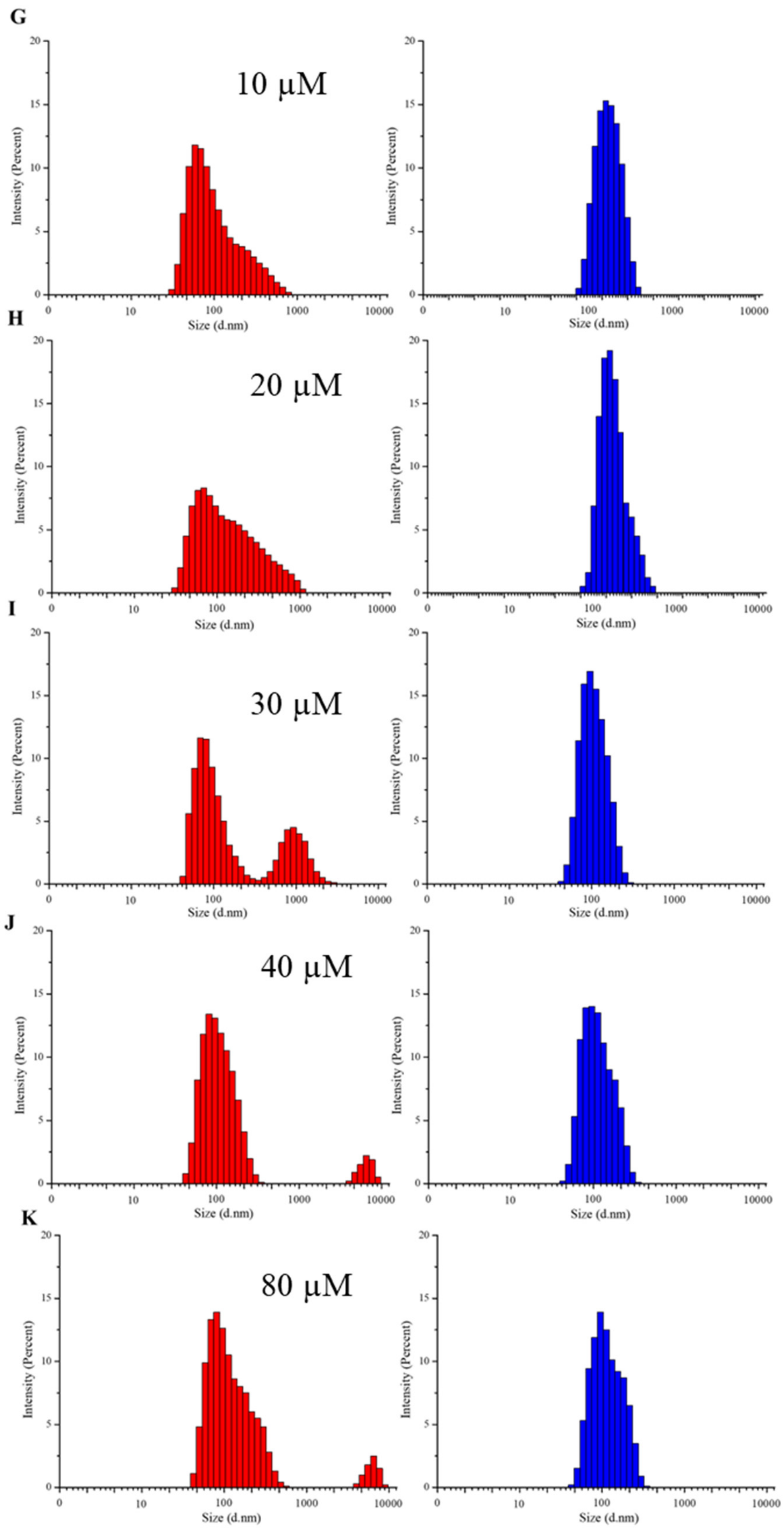


**Figure S4:** Polydispersity Index (PDI) Histograms of (A) POPC:POPG (3:1) immediate measurement; (B) POPC:POPG (3:1) measurement after 30 minutes; (C) to (K) LyeTx I (red panel) and LyeTx I-b (blue panel) at concentrations of 8-80 µM for both.

**Table S2.** Transition temperature ($T_m$) and transition enthalpy change ($\Delta_{trans}H$) values for the DMPC:DMPG (3:1) and DMPC:Chol (3:1) LUVs in the presence of both peptides. The results are presented as a function of peptide concentration.

| $T_m$ (°C) / $\Delta_{trans}H$ (kJ.mol$^{-1}$) | | | | |
|---|---|---|---|---|
| [Peptide] / μM | DMPC:DMPG (3:1) | | DMPC:Chol (3:1) | |
| | LyeTx I | LyeTx I-b | LyeTx I | LyeTx I-b |
| 0 | 23.1 / 24.5 | 23.1 / 24.5 | 22.8 / 19.5 | 22.8 / 19.5 |
| 4 | 21.7 / 23.0 | 22.1 / 22.6 | 22.2 / 19.0 | 22.7 / 19.4 |
| 8 | 20.4 / 22.0 | 20.9 / 21.5 | 21.2 / 17.5 | 22.3 / 18.5 |
| 16 | 19.3 / 20.5 | 19.3 / 19.2 | 20.7 / 16.3 | 21.3 / 18.4 |
| 32 | 18.7 / 20.2 | 18.6 / 19.0 | 19.9 / 16.0 | 21.2 / 17.5 |

Estimated uncertainties in triplicate is ± 0.2°C for $T_m$ and ± 0.5 kJ.mol$^{-1}$ for $\Delta_{trans}H$
